# Selection and Characterization of Single Chain Antibody Fragments Specific for Hsp90 as a Potential Cancer Targeting Molecule

**DOI:** 10.3390/ijms160819920

**Published:** 2015-08-21

**Authors:** Edyta Petters, Aleksandra Sokolowska-Wedzina, Jacek Otlewski

**Affiliations:** 1 Faculty of Biotechnology, Department of Protein Engineering, University of Wroclaw, Joliot-Curie 14a, 50-383 Wroclaw, Poland; E-Mails: edyta_petters@op.pl (E.P.); aleksandra.sokolowska-wedzina@uwr.edu.pl (A.S.-W.); 2 Wroclaw Research Centre EIT+, Stablowicka 147, 54-066 Wroclaw, Poland

**Keywords:** antibody fragments, cancer marker, Heat Shock Protein 90, phage display

## Abstract

Heat shock proteins play an essential role in facilitating malignant transformation and they have been recognized as important factors in human cancers. One of the key elements of the molecular chaperones machinery is Hsp90 and it has recently become a target for anticancer therapeutic approaches. The potential and importance of Hsp90-directed agents becomes apparent when one realizes that disruption of Hsp90 function may influence over 200 oncogenic client proteins. Here, we described the selection and characterization of Hsp90-specific antibody fragments from commercially available Tomlinson I and J phage display libraries. The affinities of Hsp90-binding scFv variants were measured using SPR method. Then, based on the best clone selected, we performed the affinity maturation procedure and obtained valuable Hsp90-specific clones. The selected binders were expressed and applied for immunostaining, ELISA and SPR analysis using model cancer cell lines. All performed experiments confirmed the ability of selected antibodies to interact with the Hsp90. Therefore, the presented Hsp90-specific scFv, might be a starting point for the development of a novel antibody-based strategy targeting cancer.

## 1. Introduction

Heat shock protein 90 (Hsp90) is an evolutionary conserved protein that accounts for 1%–2% of total cellular proteins and is essential for cell viability. Hsp90 is ATP-dependent molecular chaperone that assists client proteins in proper folding [1]. There are over 200 protein substrates of Hsp90 [2], many of which are the key factors in cancer development and progression, including tyrosine kinases (e.g., Src), serine-threonine kinases (e.g., Raf-1, AKT), cell cycle kinases (e.g., Wee1, POLO-1) [3,4], transcription factors (such as HIF-1), steroid receptors (e.g., estrogen, androgen, progesterone or glucocorticoid) and non-steroid receptors (e.g., HER2), as well as mutated forms of p53 [4,5]. Hsp90 protein is commonly overexpressed in a wide variety of human cancers, where it helps cells to tolerate imbalanced signaling caused by oncoproteins, therefore supporting the malignant transformation of tumor cells [4,6].

Hsp90 is one of the key players in breast carcinogenesis. It was shown that single nucleotide polymorphism within Hsp90α gene (*Gln488His*) is associated with a higher risk of breast cancer [7]. Zagouri and co-workers reported the gradual increase in Hsp90 expression all along the continuum of breast ductal lesions [8]. They observed a significant Hsp90 upregulation in the invasive ductal carcinoma (IDC). Moreover, it was found that within IDC a higher grade, larger tumor size, higher estrogen receptor expression and HER2 presence correlated with higher Hsp90 expression [8]. The role of Hsp90 has been proposed as a component of a mechanism through which breast cancer cells become resistant to increased cellular stress [9]. However, the level of Hsp90 varies among breast cancer subtypes. For example, triple negative breast cancer (TNBC), defined by the lack of expression of estrogen, progesterone and HER2 receptors, exhibited decreased cellular Hsp90 expression [8,9].

There are two isoforms of cellular Hsp90, referred to as Hsp90α and Hsp90β [10]. Hsp90α isoform can be secreted extracellularly (eHsp90α) [11,12,13], and it was shown to be associated with several physiological and pathological processes including wound healing [14], neuronal cell migration [15], angiogenesis, and cancer development [13,16,17,18,19]. Recent studies revealed the involvement of eHsp90α in migration, invasion and metastasis of many tumor cell types. Importantly, it was shown that the antibody or impermeable inhibitor of eHsp90α can efficiently suppress tumor metastasis in mouse models [13,16,20,21]. Furthermore, the level of eHsp90α in blood positively correlates with tumor malignancy in clinical cancer patients [13,22]. It was also reported that eHsp90α interacts with matrix metalloproteinase MMP2 and MMP9, mediators of tissue invasion and metastasis [11,18,21]; HER2, protein involved in cellular proliferation, differentiation and migration [23,24]; and LPR-1/CD91, protein implicated in cell migration [14,25].

In the last 20 years many advanced anticancer therapeutics targeting Hsp90 have been developed. Most of them are based on low-molecular weight drugs (e.g., geldanamycin and its derivatives, synthetic compounds) blocking the intracellular ATPase activity of Hsp90 and thus suppressing multiple oncogenic pathways of cancer development [26,27,28,29]. Recently, alternative therapeutic approaches have arisen (compounds with 7-azapteridine ring system), which aim in disruption of multiprotein HOP complex that consist of Hsp90 and co-chaperones [30]. At least 13 Hsp90 inhibitors are currently under clinical evaluation [31]. Particularly, pharmacological inhibition of Hsp90 has shown positive results in the breast cancer treatment. The highest clinical activity has been observed for HER2-positive metastatic breast cancer. It was suggested that Hsp90 inhibitors may play a role in the treatment of triple negative and aromatase inhibitor-resistant breast cancer subtypes [31]. Although, there are still no Hsp90-targeting drugs approved for clinical use, and undesirable adverse effects still occasionally appear in patients treated with new Hsp90 inhibitors [31,32]. In the light of recent studies, there is still a need for the development of a new class of cell-impermeable inhibitors, such as antibodies, which would specifically block the extracellular functions of eHsp90.

In this report, we show isolation, affinity maturation and characterization of scFvs specific to Hsp90α. The binding variants were selected from Tomlinson I and J libraries, which are commercially available sources of scFv with randomized residues in CDR2 and CDR3 regions and were proved to be highly functional [33,34,35,36]. Additionally, we confirmed *in vitro* reactivity and specificity of isolated antibodies by using them for ELISA, SPR analysis and staining of human breast cancer cell lines MDA MB 453 and MDA MB 231.

## 2. Results

### 2.1. Selection of Hsp90-Specific Antibody Fragments

Two commercially available scFv libraries, Tomlinson I and J, were used in phage display experiments as a potential source of Hsp90 binding clones. To avoid ligand modification, we decided to immobilize Hsp90 directly on the surface of immunotubes. Phage particles displaying scFv proteins were rescued from *E. coli* TG1 and used for panning against the antigen.

**Figure 1:**
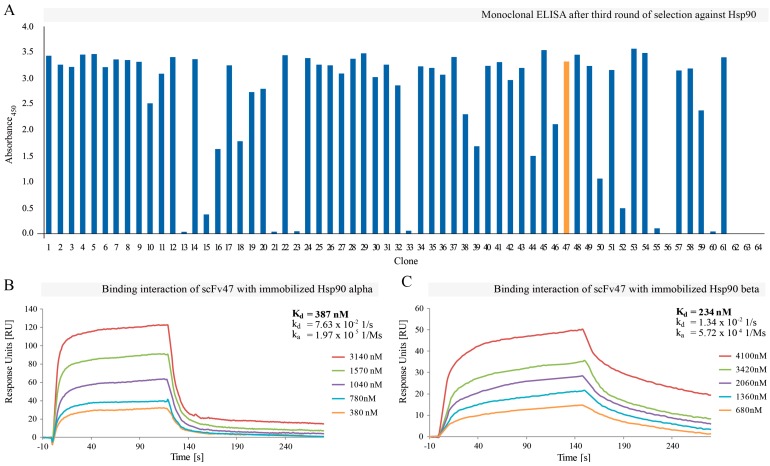
The results of phage display selection against Hsp90α. (**A**) Monoclonal ELISA for 64 scFv clones selected after the third round of panning against Hsp90α. Bacterial supernatants containing soluble antibody fragments were applied onto the target protein immobilized directly on the plastic surface of 96-well Nunc MaxiSorp^®^ plate. The binding clones were detected with the use of monoclonal mouse antibody 9E10. Of all clones, 51 showing the highest absorption at 450 nm were selected for further analysis by SPR on Biacore^®^ 3000. The best clone scFv47 is highlighted with orange; (**B**,**C**) SPR response curves for measurements of scFv47 affinity for Hsp90. Pure scFv47 in five different concentrations was injected on Hsp90 isoform α (**A**) or β (**B**) immobilized on CM5 sensor chip. The calculations of kinetic constants were done with the use of BIAevaluation 4.1 software.

After the third round of selection, we conducted monoclonal ELISA and we screened 64 individual scFv clones for binding to the target molecule. The assay showed that most of the investigated proteins exhibited some preference for Hsp90 (Figure 1A). Among them, 51 demonstrated the highest absorption signal and were employed for preliminary surface plasmon resonance (SPR) screening. The selected scFv fragments contained in bacterial supernatants were verified for binding to the Hsp90α immobilized on the CM5 sensor chip. Overall, 25 of them showed promising binding profile and were subsequently sequenced. The analysis of the sequencing results revealed no sequence identity among all clones examined, although there were some evident preferences for particular amino acid at given positions. For example, T or S was highly favored at the position 50 in HCDR2 and there were clear preferences for T, S and Y at the positions 95/96, 97 and 98 of HCDR3, respectively (data not shown). The amino acid preferences were more explicit for randomized positions in Tomlinson I library (DVT randomization scheme) than for Tomlinson J where NNK randomization was applied. Next, all 25 clones were overexpressed in bacteria, purified on Ni-NTA resin and subjected to the affinity measurements on Biacore^®^ 3000. The estimated *K*_D_ values of tested scFv fragments were mostly in the micromolar range and the highest affinity for Hsp90α was observed for scFv47 clone and was equal to 387 nM (Figure 1B).

In the next step, we decided to check if scFv47 clone was able to discriminate between Hsp90 isoforms and whether it was specific exclusively to the isoform α of this chaperone protein. We measured the affinity of scFv47 for Hsp90β in the analogical manner, as previously for Hsp90α. Surprisingly, the *K*_D_ of the tested antibody for isoform β (234 nM, Figure 1C) was 1.5-fold lower than the dissociation constant for isoform α, indicating even stronger interaction with Hsp90β.

### 2.2. Affinity Maturation of Selected scFv47

The second-generation phage display library was constructed on the basis of scFv47 clone to enable selection of antibody variants with improved binding affinities for target Hsp90α protein. As the parental clone was derived from Tomlinson I library, where the sequence diversity was introduced into HCDR2/LCDR2 and HCDR3/LCDR3 loops [37], we chose unrandomized HCDR1/LCDR1 regions to introduce the diversity in our affinity maturation library, leaving the remaining scFv47 sequence unmodified. The construction of the library, hereinafter called AM47 (affinity maturation of scFv47 clone), yielded 4 × 10^7^ individual variants. Sequencing of pIT2 plasmids from 40 distinct clones allowed to determine the 90% correctness of the library at the nucleotide level. It also indicated complete randomization at all six selected positions and showed no alterations in CDR2 and CDR3 loops (data not shown).

**Figure 2:**
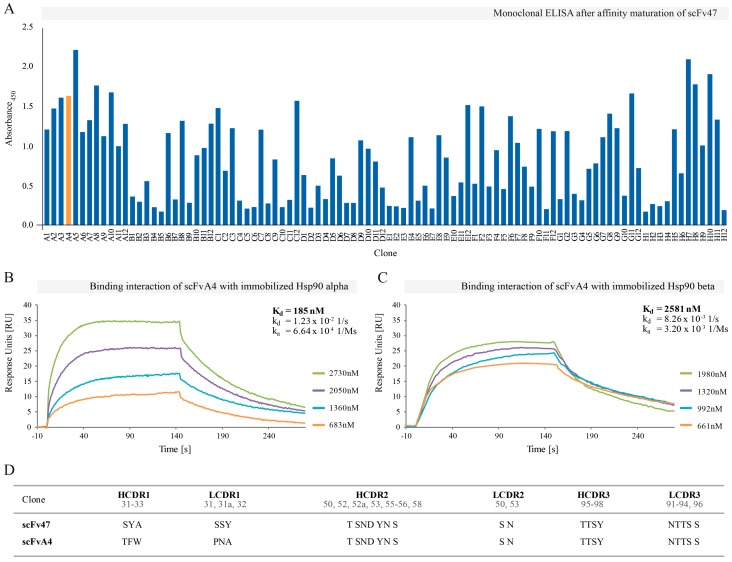
The results of affinity maturation for scFv47 clone. (**A**) Monoclonal ELISA for scFv clones selected after the second round of panning against Hsp90α. Of all clones, 35 were selected for further analysis by SPR on Biacore^®^ 3000. The best clone scFvA4 is highlighted with orange; (**B**,**C**) Binding curves for scFvA4 clone against Hsp90α and Hsp90β, respectively. Varied concentrations of purified protein were injected on two CM5 sensor chips immobilized with Hsp90 isoforms. The BIAevaluation software was used for calculations of kinetic constants; (**D**) The amino acids selected at CDR1, CDR2 and CDR3 of parental and affinity matured clones.

The panning procedure was modified to favor selection of scFv variants with improved *k*_off_ values. Therefore, we run so-called “off-rate selection”, wherein for the second round of panning we used five molar excess of soluble Hsp90α. This strategy enabled the enrichment of slower dissociating clones and led to the isolation of 35 positive variants (Figure 2A). The best clone selected, scFvA4, exhibited *K*_D_ of 185 nM (Figure 2B). Although the improvement in *K*_D_ was not significant (two-fold), the scFvA4 showed over sixfold lower *k*_off_ in comparison with parental scFv47. Moreover, we observed 10-fold weaker binding affinity of scFvA4 to Hsp90β (Figure 2C). Such result could be beneficial for future therapeutic applications, as cancer cells express and constitutively secrete primarily the isoform α of Hsp90 [38,39]. In addition, we compared our selected antibody fragments with commercially available anti-Hsp90 antibody using SPR method. We injected the same concentration of antibodies/scFvs on Hsp90α immobilized on sensor chip. We observed similar binding profiles of scFvA4 and commercial antibody (Figure S1).

### 2.3. Interaction of Antibody Fragments with Hsp90 in Breast Cancer Cell Lines

**Figure 3:**
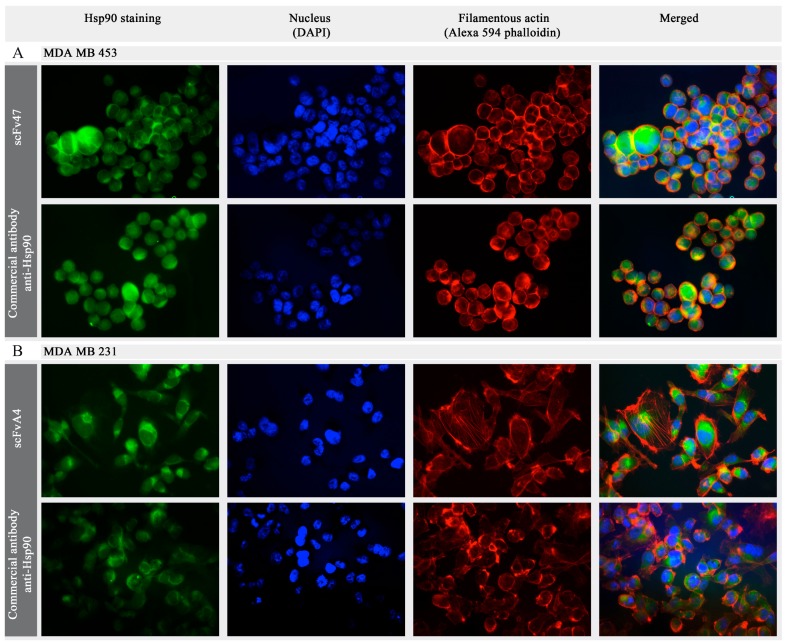
The immunofluorescence detection of intracellular Hsp90 in fixed and permeabilized. (**A**) MDA MB 453 cell line; and (**B**) MDA MB 231 cell line. The intracellular Hsp90 was stained green either by scFv47-FITC, scFvA4-FITC or anti-Hsp90/secondary goat anti-mouse Alexa 488-conjugated antibodies. Nuclei were stained with DAPI (blue) and the cytoskeletal actin filament network was visualized with Alexa 594 phalloidin (red). Original magnification ×20, for all panels.

The immunofluorescence experiments were preformed to test the ability of selected scFvs to specifically recognize Hsp90α in the cell model. We used two human breast cancer cell lines, MDA MB 435 and MDA MB 231, as Hsp90-expressing examples [19,40,41]. To visualize the scFv47 and scFvA4 binding to Hsp90, we conjugated those proteins to fluorescein isothiocyanate (FITC). As a positive control, commercially available mouse antibody specific for Hsp90 was used. Additionally, the nuclei were stained with DAPI and the cytoskeletal actin filament network was visualized with Alexa 594 phalloidin (Figure 3).

Both of the tested scFvs recognized the cytoplasmic pool of Hsp90 as demonstrated by immunofluorescence after fixation and permeabilization of cells, and showed similar Hsp90-staining pattern, comparable with the commercially available antibody used as a control.

### 2.4. Binding of Specific scFv to Extracellular Pool of Hsp90

**Figure 4:**
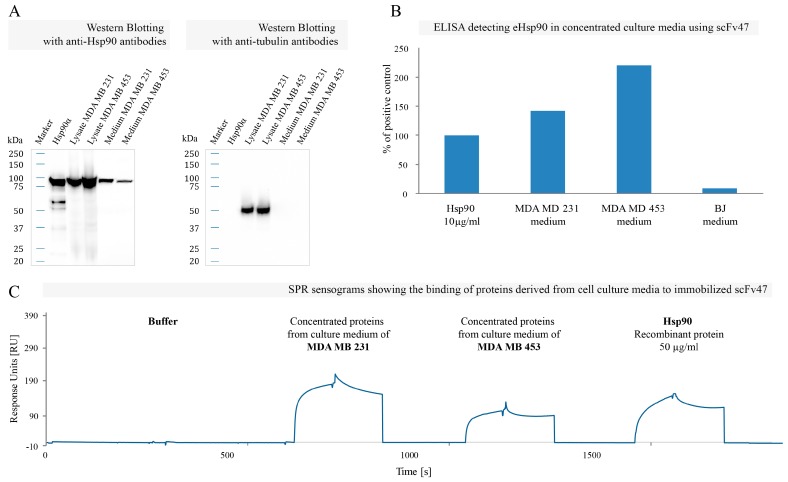
Interaction of scFv47 with eHsp90: (**A**) Western blot analysis of concentrated serum-free media derived from MDA MB 231 and MDA MB 453 cell cultures using specific anti-Hsp90 antibodies. Cell lysates containing cytoplasmic Hsp90 and recombinant Hsp90α served as positive controls; (**B**) The representative ELISA using scFv47 to detect eHsp90 in concentrated culture media derived from MDA MB 231, MDA MB 453 and BJ cells. The plate was coated with scFv47 and cell medium was applied. Detection of bound Hsp90 was performed using commercially available mouse anti-Hsp90 followed by anti-mouse-HRP antibody. Absorbance value from the controls with no antigen was subtracted from the obtained results, which were subsequently normalized as a fraction of absorbance from the wells where 10 µg/mL of recombinant Hsp90α was loaded (positive control); (**C**) The SPR sensograms showing the binding of Hsp90α to immobilized scFv47. After 38 hours of culturing MDA MB 231 and MDA MB 453 cells (in serum-free DMEM) media were collected, concentrated 100 times, buffer exchanged and injected on a sensor chip coated with *ca*. 1500 RU scFv47. Fifty micrograms per milliliters of the recombinant Hsp90α was injected as a positive control.

Based on previous studies that reported the secretion of Hsp90 by human breast cancer cells MDA MB 231 [42] and MDA MB 453 [21,41], lysates of MDA MB 231 and MDA MB 453 cells as well as concentrated supernatants derived from the serum-free cell medium upon 38 h of culture were analyzed by Western blotting using commercially available anti-Hsp90 antibody. As shown in Figure 4A, Hsp90 was present not only in lysates (cytoplasmic Hsp90) but also in the media (extracellular Hsp90), indicating that both breast cancer cell lines tested secreted Hsp90. The absence of tubulin in the medium fraction (Figure 4A) confirmed that there was no contamination with the intracellular components during the experimental procedure.

In order to show that eHsp90 could be efficiently recognized by our specific antibody fragments, we subjected the concentrated media from cell culture to ELISA. In this experiment scFv47 was immobilized on the plate and used as an eHsp90 capture antibody fragment. Concentrated media derived from the 72-h cell culture, as well as recombinant Hsp90α were applied on the plate and detection of Hsp90 bound to scFv47 was performed using commercially available mouse anti-Hsp90 antibody followed by anti-mouse-HRP antibody. Positive signal from extracellular Hsp90 was detected in both breast cancer cell lines, whereas low signal value was observed for non-cancer human skin fibroblast cell line (BJ) (Figure 4B). Importantly, no significant signal was detected when analyzing culture media using scFvC1 specific for Fibroblast Growth Factor Receptor 1 (FGFR1) selected in our lab from the same library (Figure S2).

In addition, we confirmed the ability of scFv47 to bind extracellular pool of Hsp90 by SPR technique. Concentrated serum-free media derived from the 38-hour culture of MDA MB 231 and MDA MB 453 breast cancer cell lines were subjected to buffer exchange and injected on a sensor chip coated with scFv47. As shown in Figure 4C, SPR sensograms confirmed interaction between immobilized scFv47 and both MDA MB 231 and MDA MB 453 cell media. Moreover, the binding specificity observed for both cell lines was similar to results obtained for recombinant Hsp90α injected as a positive control (Figure 4C). Thus, our data strongly indicate that observed interaction is due to the binding of scFv47 to eHsp90 secreted by both cancer cell lines to culture media.

## 3. Discussion

The important role of Hsp90 in malignancy is to support cancer cells in overcoming environmental stresses. This function is mediated by Hsp90 capability to improve the stability of its client oncoproteins. Inhibition of intracellular function of Hsp90 results in combinatorial disruption of multiple oncogenic signal transduction pathways. Thus, Hsp90 became a viable target for antitumor drug development. Until now, several low-molecular weight intracellular Hsp90 inhibitors have entered the clinical trials. Unfortunately, no Hsp90 inhibitor has been FDA-approved to date, also due to the observed side effects of tested agents [43,44,45]. Therefore, there is a need to search for a new therapeutic solution that may enhance efficacy of Hsp90 inhibitors. According to recent studies, extracellularly expressed Hsp90α could be a valuable target for anti-cancer therapy. It was shown that eHsp90α is particularly important in tumor metastasis and angiogenesis [15,18,19,39]. The *in vivo* studies revealed that monoclonal antibody 4C5 significantly inhibits formation of metastatic breast cancer cell deposits in mice [21]. In addition, many types of tumor cells secrete Hsp90 constitutively to promote cell motility and invade the tissue, whereas normal cells secrete Hsp90 only in response to tissue injury [19,39]. Targeting extracellular Hsp90 with new generation inhibitors, which would be unable to enter the cells, could be used to treat cancer metastasis and improve selectivity of Hsp90-targeted anticancer therapy.

The aim of this study was to obtain Hsp90-specific scFv as a potential tool for anticancer therapy. We demonstrated successful selection and affinity maturation of single chain antibody fragments towards Hsp90α isoform. We used commercially available Tomlinson I and J libraries as a source of high-affinity binders. By modifying the standard phage display selection protocol we were able to obtain scFv molecules showing favorable binding both to recombinant Hsp90α and recombinant Hsp90β. Moreover, we employed affinity maturation procedure with subsequent “off-rate selection” to successfully increase the *k*_off_ value of selected scFv variant and improve its affinity to the target protein Hsp90α. We applied different techniques to demonstrate functionality of selected antibody fragments in detection of Hsp90 present in human breast cancer models. First, we used immunofluorescence to show interactions of selected scFv47 and scFvA4 with cytoplasmic pool of Hsp90 protein using permeabilized cancer cells. Next, we confirmed by Western blotting previously reported secretion of Hsp90 by MDA MB 231 and MDA MB 453 cells, and then we used one of our scFvs to detect eHsp90 in culture media by ELISA. Eventually, we demonstrated the ability of scFv47 to bind extracellular pool of Hsp90 present in cell culture media by SPR technique. In conclusion, our results clearly demonstrate that scFvs selected in this study effectively recognize cytoplasmic as well as extracellular Hsp90 in breast cancer cellular models.

Selected single-chain Fv modules may then serve as a building block for further engineering of improved formats of immunoproteins, for example, full membrane-impermeable antibody, targeting extracellular Hsp90. Such antibody-based inhibitor could be useful in treatment of cancers whose invasiveness and metastasis depend on eHsp90 secretion, including breast cancer [11,13,21,40,42,46], melanoma [12,46], fibrosarcoma [11,42], colorectal cancer [22], bladder cancer [46], prostate cancer [46], and glioblastoma [42]. Therefore, we believe that the new Hsp90 binder selected within this study is a good starting point for the development of a novel antibody-based strategy specifically targeting cancer metastasis.

## 4. Experimental Section

### 4.1. Selection Experiments Based on Tomlinson I and J Libraries

The panning experiments were conducted according to manufacturer’s instruction available online [37], with some modifications. In the first round of selection the immunotubes were coated overnight with 4 mL of 50 μg/mL recombinant Hsp90α (StressMarq Biosciences Inc., Victoria, BC, Canada) in PBS. In the second and third round of selection the antigen concentrations were half decreased. Incubation times for binding of phage particles to the target molecules were 2 h in round 1 and 1 h in rounds 2 and 3 (half the time with rotation, the other half without). Unbound phage particles were washed away with PBS-0.1% Tween 20 using 5 washes in the first round, 10 in the second and 20 in the third. Each time the last wash was PBS.

### 4.2. Affinity Maturation: Library Construction and Panning

In the Tomlinson libraries, the side chain diversity was incorporated at positions in the CDR2 and CDR3 regions. Thus, the affinity maturation library was constructed by randomizing amino acid residues in the CDR1 of both heavy and light chain, as previously described [47]. Diversification of side chains was introduced by series of Polymerase Chain Reactions, using degenerate primers (NNK). The randomized DNA fragments were subjected to enzymatic digestion and ligated into pIT2 vector. Desalted ligation mixture was then electroporated into *E. coli* TG1 bacteria. The size of the library was estimated by serial dilutions of transformed cells and sequencing of randomly selected clones allowed to assess the quality of the library, *i.e.*, the percentage of randomized positions.

In the first round of panning, phage particles were incubated with 10 µg/mL of recombinant Hsp90α for 2 h and unbound phage particles were washed away with 10 washes of PBS-0.1% Tween 20. In the second round, the antigen concentration, the incubation time, and the number of washes were reduced by half. To promote selection of slowly dissociating clones, in the second round 5-fold molar excess of recombinant Hsp90α was added to the solution for additional 1 h.

### 4.3. Monoclonal ELISA of Soluble Antibody Fragments

Monoclonal ELISA was used for initial screening of scFv clones. Bacterial supernatants containing soluble myc-tagged scFv fragments were obtained as previously described [47]. Briefly, individual colonies were picked into 200 µL 2× TY/100 µg Ampicillin/0.1% glucose in 96-well plates and incubated for 3 h at 37 °C with shaking. Then, 1 mM of IPTG was added to induce scFv expression and the cultures were grown at 30 °C overnight. Nunc MaxiSorp^®^ plates (Thermo Scientific, Waltham, MA, USA) were coated overnight with recombinant Hsp90α in the same buffer and at the same concentration as for the selection. Bacterial supernatants were added do immobilized antigen and incubated for 1 h at rt. Unbound scFv were washed away and bound antibody fragments were detected with monoclonal mouse antibody 9E10 (Sigma-Aldrich, St. Louis, MO, USA). Horseradish peroxidase conjugated goat anti-mouse IgG (Sigma-Aldrich, St. Louis, MO, USA) was used as a secondary antibody, followed by addition of TMB substrate solution (Sigma-Aldrich, St. Louis, MO, USA) for assay developing. The reaction was stopped by addition of 1 M H_2_SO_4_. The absorbance values were measured at 450 nm.

### 4.4. SPR (Surface Plasmon Resonance) Screening of Selected Clones

Variants of antibody fragments positive in ELISA assay were further evaluated using SPR screening on Biacore^®^ 3000 instrument (GE Healthcare, Little Chalfont, UK). Bacterial supernatants (the same as used for ELISA) were filtered through 0.22 µm filters and analyzed for ligand binding on CM5 sensor chip coated with covalently immobilized recombinant Hsp90α isoform (StressMarq Biosciences Inc., Victoria, BC, Canada) at about 3500 RU.

### 4.5. Soluble scFv Expression and Purification

Recombinant scFv proteins in fusion with 6× HIS tag were expressed in *E. coli* TG1 cells and purified form bacterial periplasm using Ni-NTA Agarose (Qiagen, Hilden, Germany). Briefly, bacterial cells were ruptured with osmotic shock buffer (30 mM Tris, 20% sucrose, 1 mM EDTA, pH 7.0), centrifuged and scFv-containing fraction was dialyzed to PBS, 500 mM NaCl. Then, the solution was incubated with the resin for 1 h at 4 °C. The unbound proteins were removed by washing the column with PBS, 500 mM NaCl, and scFv were eluted with PBS, 500 mM NaCl, 300 mM imidazole and dialyzed to PBS, 1 mM EDTA. Purified scFv proteins were analyzed by Size-Exclusion Chromatography on Superdex 75 resin and fractions containing pure protein of the correct molecular mass were collected. Then, the scFv concentration was estimated with NanoDrop spectrophotometer (Thermo Scientific, Waltham, MA, USA).

### 4.6. Affinity Measurements by SPR

Pure antibody fragments were serially diluted and analyzed on Biacore^®^ 3000 instrument for binding to recombinant Hsp90α and recombinant Hsp90β (StressMarq Biosciences Inc., Victoria, BC, Canada) immobilized on CM5 sensor chip at about 3500 RU. Measurements were performed in PBS-EPN buffer (running and sample buffer; PBS, 0.005% *v*/*v* surfactant P20, 0.02% NaN_3_; pH 7.4). Fifty microliters injections of samples were made at a flow rate of 20 µL/min. Dissociation of the analyte from ligand was monitored for 2 min. For regeneration of the sensor chip surface 10 mM glycine was used. The binding curves of individual clones were analyzed using BIAevaluation 4.1 software (GE Healthcare, Little Chalfont, UK).

### 4.7. Cell Cultures

Human breast cancer cells MDA MB 435, MDA MB 231 and human skin fibroblast BJ were cultured on plastic dishes in Dulbecco’s Modified Eagle’s Medium (DMEM) containing 10% Fetal Bovine Serum (FBS) supplemented with 1× penicillin/streptomycin solution under standard conditions recommended by ATCC.

### 4.8. Immunofluorescence

Human breast cancer cells MDA MB 435 and MDA MB 231 were grown on cover slips under standard conditions. Then, at room temperature, the cells were washed twice with pre-warmed PBS, fixed with 4% formaldehyde for 15 min and permeabilized by 1% Triton V-100 for 30 min before blocking with 100% FBS for 15 min. After that, appropriate concentrations of FITC-conjugated scFv or commercially available positive control primary mouse anti-Hsp90 antibody (BD Biosciences, San Jose, CA, USA) were added. In case of control slides, goat anti-mouse Alexa 488-conjugated antibodies (Invitrogen, Thermo Scientific, Waltham, MA, USA) were used secondary reagents. Additionally, phalloidin-Alexa 594 and DAPI-containing Vectashield^®^ (Vector Laboratories, Burlingame, CA, USA) reagents were employed to detect actin cytoskeleton and nuclei, respectively.

### 4.9. Preparation of Concentrated Cell Culture Media for Western Blotting and SPR Measurements

MDA MB 435 and MDA MB 231 cells were grown under standard conditions. When cells reached confluence, the initial medium was replaced by a serum-free medium (DMEM without FBS) and the cells were incubated 38 h. Then, the supernatants derived from the same number of cultured cells were collected. Media were filtrated through 0.22 µm filters and concentrated 100-fold using Centriprep Centrifugal Filter Unit with a membrane NMWL of 10 kDa (Merck Millipore, Billerica, MA, USA). The concentrated supernatants were used for Western blotting. For SPR measurements additional buffer exchange to PBS-EPN was performed using Zeba Spin Desalting Columns (Thermo Scientific, Waltham, MA, USA).

### 4.10. Preparation of Concentrated Cell Culture Media for ELISA

MDA MB 435 and MDA MB 231 and BJ cells were grown under standard conditions. When cells reached confluence, the media derived from the same number of cultured cells were collected. Media were filtrated through 0.22 µm filters and concentrated 50-fold using Centriprep Centrifugal Filter Unit with a membrane NMWL of 10 kDa.

### 4.11. Western Blotting

Equal volumes of the concentrated cell culture media were separated on SDS-PAGE 4%–20% gradient gel (Pierce, Thermo Scientific, Waltham, MA, USA) and electrotransferred to a PVDF membrane (Immobilon^®^, Merck Millipore, Billerica, MA, USA). The membrane was incubated with the anti-Hsp90 mouse antibody or anti-tubulin mouse antibody (Sigma-Aldrich, St. Louis, MO, USA) followed by incubation with anti-mouse HRP-conjugated secondary antibodies. The blot was developed with the ECL reagent (Pierce, Thermo Scientific, Waltham, MA, USA) according to the manufacturer’s instructions.

### 4.12. ELISA for Hsp90 Detection in Cell Culture Media

Wells of 96-well Nunc MaxiSorp^®^ plates were coated with 50 µg/mL of scFv47 in PBS overnight at RT and then were blocked with 2% Marvel milk in PBS (2% MPBS) for 2 h at RT. Concentrated cell culture media with 2% Marvel milk were loaded and incubated for 1 h 40 min at RT. Unbound proteins were washed away and bound Hsp90 was detected with anti-Hsp90 antibody. Further steps were performed as described in Section 4.3.

### 4.13. SPR Measurements of Hsp90 Binding to Immobilized scFv47

Protein–protein interactions of scFv47 and concentrated proteins from culture media or recombinant Hsp90α were subjected to SPR analysis using Biacore^®^ 3000 instrument. scFv47 in 10 mM sodium acetate buffer, pH 4.0 was covalently coupled on CM5 sensor chip surface using a standard amine coupling protocol. scFv47 was immobilized at about 1500 RU. Measurements were performed in PBS-EPN buffer. A 20 µL aliquot of analyte was injected at a flow rate of 10 µL/min. Dissociation of the analyte from ligand was monitored for 2 min. For regeneration of the sensor chip surface 10 mM NaOH was applied. The binding curves were analyzed using BIAevaluation 4.1 software.

## 5. Conclusions

Here we presented successful selection and affinity maturation of single chain antibody fragments against Hsp90α isoform. Using different approaches we confirmed the ability of selected antibodies to interact with the Hsp90. We demonstrated that scFvs selected in this study effectively recognize cytoplasmic as well as extracellular Hsp90 in breast cancer cellular models.

## Supplementary Materials

Supplementary materials can be found at http://www.mdpi.com/1422-0067/16/08/19920/s1.
