# Supplementary material for: Selection and Characterization of Single Chain Antibody Fragments Specific for Hsp90 as a Potential Cancer Targeting Molecule
Source: Int J Mol Sci. 2015 Aug 21;16(8):19920–35. doi: 10.3390/ijms160819920 (PMC4581332; doi:10.3390/ijms160819920)
Supplement: Supplementary file 1 [file ijms-16-19920-s001.pdf]

Supplementary Information

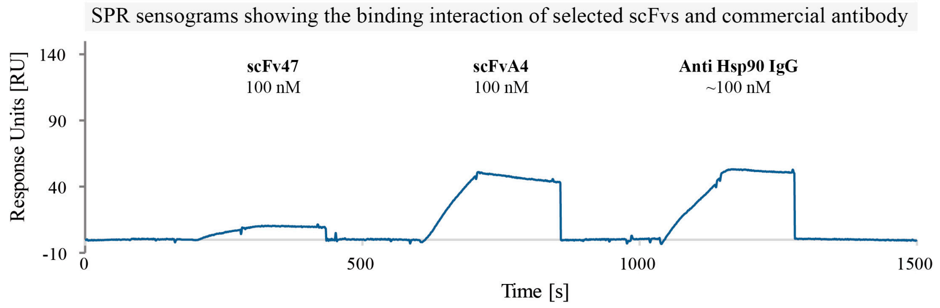

Figure S1. SPR sensograms showing the binding interaction of selected scFv47, scFvA4 and commercial anti-Hsp90 antibody at the concentration of 100 nM with recombinant Hsp90α (Abcam) immobilized on CM4 sensor chip at about 7000 RU. SPR measurements were performed according to the protocol described in Experimental Section 4.13.

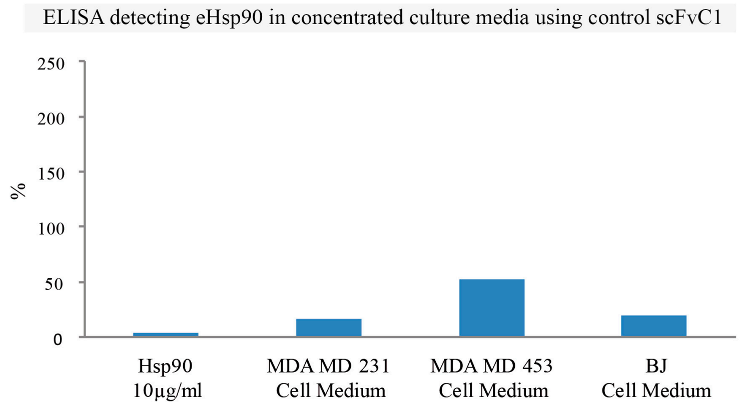

Figure S2. The representative ELISA using control scFvC1 (specific for FGFR1) for detection of eHsp90 in concentrated culture media derived from MDA MB 231, MDA MB 453 and BJ cells. Detection of Hsp90 was performed using commercially available mouse anti-Hsp90 followed by anti-mouse-HRP antibody. Absorbance value from the controls with no antigen was subtracted from the obtained results, which were subsequently normalized as a fraction of absorbance from the wells where 10 µg/mL of recombinant Hsp90α was loaded (positive control).
